# Expanding the epitranscriptome: Dihydrouridine in mRNA

**DOI:** 10.1371/journal.pbio.3001720

**Published:** 2022-07-20

**Authors:** Sameer Dixit, Samie R. Jaffrey

**Affiliations:** Department of Pharmacology, Weill-Cornell Medical College, Cornell University, New York, New York, United States of America

## Abstract

Nucleotide modifications can markedly influence mRNA processing and metabolism. This Primer explores two new studies, one in PLOS Biology, showing that ~130 yeast mRNAs contain dihydrouridine, a derivative of uridine. Functional studies show that dihydrouridine, in some cases, can affect mRNA splicing.

## Teaser

Nucleotide modifications can markedly influence mRNA processing and metabolism. New studies, one in *PLOS Biology*, show that approximately 130 yeast mRNAs contain dihydrouridine, a derivative of uridine. Functional studies show that dihydrouridine, in some cases, can affect mRNA splicing.

Ever since the initial transcriptome-wide maps of m^6^A established that m^6^A is widespread and can regulate specific cohorts of mRNAs [1], many have asked whether other nucleotide modifications are found in mRNA. Besides classical RNA editing of adenosine to form inosine, pseudouridine and 5-methylcytidine have been identified in mRNA, although these modifications are relatively rare compared to m^6^A [2,3]. Other modifications have also been described as widespread in mRNA, including *N*^1^-methyladenosine (m^1^A), 2-*O*-methyl modifications, and *N*^7^-methylguanosine (m^7^G), but the validity of these maps and their putative high abundance in mRNA has been challenged based on technical problems with the mapping methods [4].

Although discovering modifications in mRNA might seem simple—mass spectrometry of hydrolyzed mRNA should reveal all the modifications—mRNA preparations are invariably contaminated with small amounts of tRNA or rRNA [5]. Without rigorous standards to quantify contamination levels, it is hard to know if a modified nucleotide detected by mass spectrometry truly derives from mRNA. Mapping methods can provide confidence that the modification is in mRNA since the precise site in each mRNA can be identified and validated biochemically.

**Fig 1:**
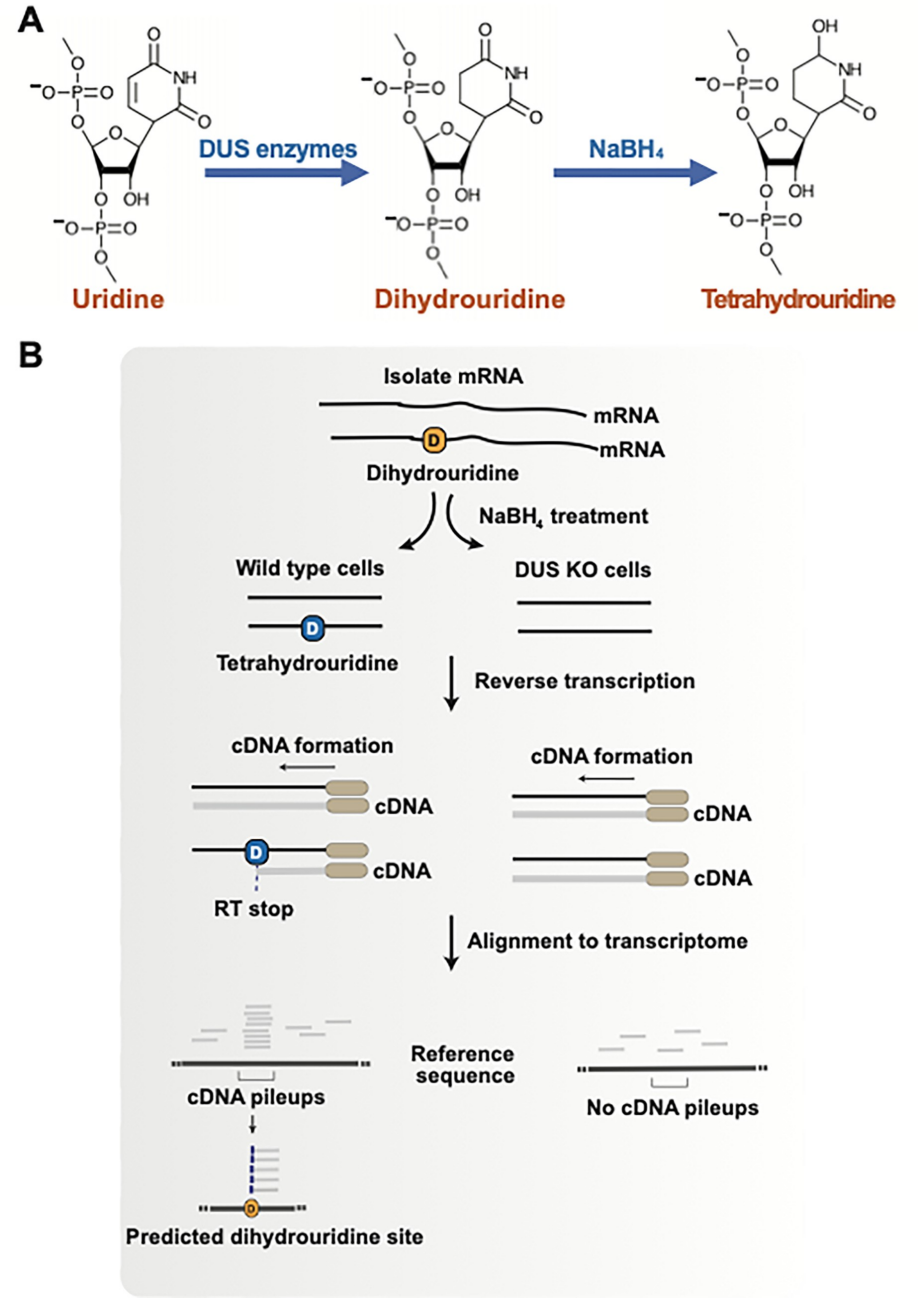
Transcriptome-wide mapping of dihydrouridine with D-seq. **(**A) DUS enzymes catalyze the reduction of uridine to dihydrouridine, which comprises the loss of a double bond in the uracil base. Further chemical reduction using sodium borohydride (NaBH_4_) results in formation of tetrahydrouridine, which results in an increased frequency of reverse transcriptase terminations. (B) Protocol for dihydrouridine profiling in RNA using sodium borohydride treatment in wild-type and *DUS* KO cells. The sites of reverse transcriptase terminations are mapped. In RNA samples treated with sodium borohydride, the pattern of terminations changes, with an enrichment at sites of tetrahydrouridine. DUS, dihydrouridine synthase; KO, knockout; RT, reverse transcriptase.

Dihydrouridine is a modified form of uridine in which one of the double bonds is reduced (**Fig 1**). Dihydrouridine is normally thought of as a tRNA modification that is found in its “D-loop,” which is a stem-loop structure in tRNA that contains dihydrouridine, often abbreviated as “D.” A major hint that dihydrouridine might be in mRNA came from previous crosslinking studies that showed that the dihydrouridine synthase (DUS) enzymes can be cross-linked to mRNA transcripts [6]. Two studies have now identified dihydrouridine as a modified nucleotide in mRNA [7,8]. A major function of dihydrouridine is to influence RNA folding. The modified uracil ring in dihydrouridine causes the ribose portion of the nucleotide to adopt a different “pucker” orientation than other ribonucleotides, thus causing dihydrouridine to disrupt the precise geometry of RNA helices. Dihydrouridine also is less likely to occur in helical regions since dihydrouridine:adenosine base pairs are less stable than uridine:adenosine base pairs due to a slightly altered orientation of the dihydrouridine nucleobase. Lastly, the nucleobase in dihydrouridine is not planar, which prevents base stacking interactions that have important roles in RNA structure. Overall, dihydrouridine can bias RNA folding so that it is not found in helical regions but instead is found in loop regions of stem loop structures.

To map dihydrouridine sites, Draycott developed “D-Seq” [7]. D-Seq uses sodium borohydride, a chemical that converts dihydrouridine to tetrahydrouridine. Tetrahydrouridine impairs reverse transcription. Therefore, after sodium borohydride treatment, the RNA is reverse transcribed, and the cDNA is cloned into a next-generation sequencing library. The 3′ ends of the cDNA are then mapped to the transcriptome. As a result, “pileups” of cDNA 3′ ends that only occur in control cells and not DUS-depleted cells are expected to mark the location of dihydrouridine sites. Using D-seq, Draycott and colleagues confirmed the locations of dihydrouridine in tRNA and identified 48 novel sites in 23 different small nucleolar RNAs (snoRNAs) [7].

The authors also looked for dihydrouridine in yeast mRNA. Unlike tRNA and snoRNA, each different mRNA is present at low abundance. This means that there are only a few reverse transcription 3′ ends to map for each mRNA. As a result, distinguishing dihydrouridine-induced reverse transcriptase termination from sporadic reverse transcriptase stops is difficult. Draycott and colleagues used rigorous statistical methods along with numerous replicates to identify reverse transcription stops that recurrently occurred in control mRNA but were completely lost in the DUS knockout samples. Using this approach, the authors estimated 130 dihydrouridine sites in mRNA [7]. It should be noted that other sites might be prevalent, but mRNA abundance or possibly low stoichiometry dihydrouridine modification may have prevented their detection.

In mRNA, dihydrouridine was found in 5′ and 3′ UTRs, as well as coding sequences. Using structure-probing methods, the authors found that dihydrouridine was often found in loop regions of stem-loop structures [7]. There are 2 interpretations of these experiments, both probably true. First, these stem-loop structures might be the substrate of DUSs to form dihydrouridine. Second, dihydrouridine could bias RNA folding to ensure that dihydrouridine is in the loop region of stem-loop structures.

The authors considered several possibilities for the potential impact of dihydrouridine on mRNA. In synthetic mRNAs containing small numbers of dihydrouridine, they found no significant impact on translation using in vitro translation assays.

However, the authors found that dihydrouridine can affect splicing, particularly in the case of *RPL30*. In *RPL30*, a dihydrouridine was detected in an intron adjacent to an RNA structure that enables RPL30 to autoregulate its own splicing. In the absence of DUS enzymes, the introns were retained, suggesting that dihydrouridine is needed for the proper splicing of RPL30. Notably, the splicing of other dihydrouridine-containing RNA was not similarly affected, indicating that this dihydrouridine does not generally inhibit splicing.

Finet and colleagues described Rho-seq [8], which is similar to D-seq. Rho-seq is named for rhodoamine, which is covalently added to the dihydrouridine after the sodium borohydride step. This bulky adduct leads to efficient reverse transcriptase stalling. Finet and colleagues found 229 dihydrouridine sites in tRNA and 143 in other RNAs, of which 125 were found in protein-coding transcripts [8]. Among dihydrouridine-containing mRNAs, several of them encoded cytoskeleton-related proteins like the tubulin *nda2* and *nda3* genes. Notably, these authors mapped dihydrouridine in mammalian cells, revealing 112 mRNA sites, including *TUBA1C*, the human homolog of *nda2*. This suggests that the function of dihydrouridine in this mRNA is likely to be evolutionarily conserved.

Finet and colleagues explored the dihydrouridine in *nda2* and *nda3*, which are both required for meiotic progression. Blocking dihydrouridine formation by either depletion of DUS activity or by mutation of the uridine resulted in enhanced protein synthesis. Therefore, these authors concluded that dihydrouridine was critical for suppressing the translation of specific mRNAs [8]. Since the translational suppression effects of dihydrouridine were tested and were not seen by Draycott and colleagues, it remains unclear if the translation-suppressing function of dihydrouridine may be limited to specific transcripts.

Although there were many similarities in the patterns of dihydrouridine sites that were identified by D-seq and Rho-seq [7,8], there were also differences such as a significant enrichment of dihydrouridine in the context of a U-tract seen with Rho-seq. This may reflect the differences in the yeast species that were used (*Saccharomyces cerevisiae* in D-seq and *Schizosaccharomyces pombe* for Rho-seq). Differences in the sequencing depth of the datasets and the bioinformatic methods to identify dihydrouridine can affect which sites are called. Orthogonal biochemical assays, such as SCARLET [9], will be useful to validate these sites and determine the stoichiometry of dihydrouridine modification. Ultimately, determining if and how dihydrouridine synthesis is regulated, and whether these dihydrouridine sites have functional consequences will be important for establishing dihydrouridine as a new RNA regulatory mechanism.

## Abbreviations:

DUS: dihydrouridine synthase
m^1^A: *N*^1^-methyladenosine
m^7^G: *N*^7^-methylguanosine
snoRNA: small nucleolar RNA
